# Strong geologic methane emissions from discontinuous terrestrial permafrost in the Mackenzie Delta, Canada

**DOI:** 10.1038/s41598-017-05783-2

**Published:** 2017-07-19

**Authors:** Katrin Kohnert, Andrei Serafimovich, Stefan Metzger, Jörg Hartmann, Torsten Sachs

**Affiliations:** 1 GFZ German Research Centre for Geosciences, Telegrafenberg, 14473 Potsdam Germany; 2 National Ecological Observatory Network, Battelle, 1685 38th Street, Boulder, CO 80301 USA; 3 University of Wisconsin-Madison, Dept. of Atmospheric and Oceanic Sciences, 1225 West Dayton Street, Madison, WI 53706 USA; 4 Alfred Wegener Institute Helmholtz Centre for Polar and Marine Research (AWI), Am Handelshafen 12, 27570 Bremerhaven, Germany

## Abstract

Arctic permafrost caps vast amounts of old, geologic methane (CH_4_) in subsurface reservoirs. Thawing permafrost opens pathways for this CH_4_ to migrate to the surface. However, the occurrence of geologic emissions and their contribution to the CH_4_ budget in addition to recent, biogenic CH_4_ is uncertain. Here we present a high-resolution (100 m × 100 m) regional (10,000 km²) CH_4_ flux map of the Mackenzie Delta, Canada, based on airborne CH_4_ flux data from July 2012 and 2013. We identify strong, likely geologic emissions solely where the permafrost is discontinuous. These peaks are 13 times larger than typical biogenic emissions. Whereas microbial CH_4_ production largely depends on recent air and soil temperature, geologic CH_4_ was produced over millions of years and can be released year-round provided open pathways exist. Therefore, even though they only occur on about 1% of the area, geologic hotspots contribute 17% to the annual CH_4_ emission estimate of our study area. We suggest that this share may increase if ongoing permafrost thaw opens new pathways. We conclude that, due to permafrost thaw, hydrocarbon-rich areas, prevalent in the Arctic, may see increased emission of geologic CH_4_ in the future, in addition to enhanced microbial CH_4_ production.

## Introduction

The emission of biogenic methane (CH_4_) from arctic permafrost landscapes caused by microbial decomposition of carbon is widely discussed^1–6^. The carbon pool of arctic permafrost soils is estimated at 1300 Pg (ref. 7). Emission estimates of biogenic CH_4_ from permafrost regions vary between 32 and 112 Tg per year^8^. The production of biogenic CH_4_ in wetlands is limited to soil temperatures allowing for microbial activity. This is the case during the warm season of the year, but also at soil temperatures around 0 °C, the “zero curtain”^9^.

In addition to the biogenic CH_4_ sources, oil and gas reservoirs in the Arctic contain vast amounts of old, geologic CH_4_ (ref. 10) that can be of biogenic or thermogenic origin. Currently, in large areas this source is sealed by a cap of permafrost. Geologic CH_4_ can reach the surface if pathways in this cap exist along faults (cf. ref. 11 for lower latitudes) or form due to permafrost thawing^12, 13^. For subsea permafrost no consensus has been reached on the amount of geologic CH_4_ released from e.g. the Siberian shelf^14, 15^. In terrestrial permafrost landscapes geologic CH_4_ can reach the surface through open taliks (=thaw bulbs) below lakes^13^, or in the unfrozen areas of discontinuous permafrost, comparable to what was shown for lower latitudes^11, 16^. Until now, the occurrence of the entirety of these geologic sources in the Arctic and their contribution to the overall CH_4_ emission is uncertain.

**Figure 1:**
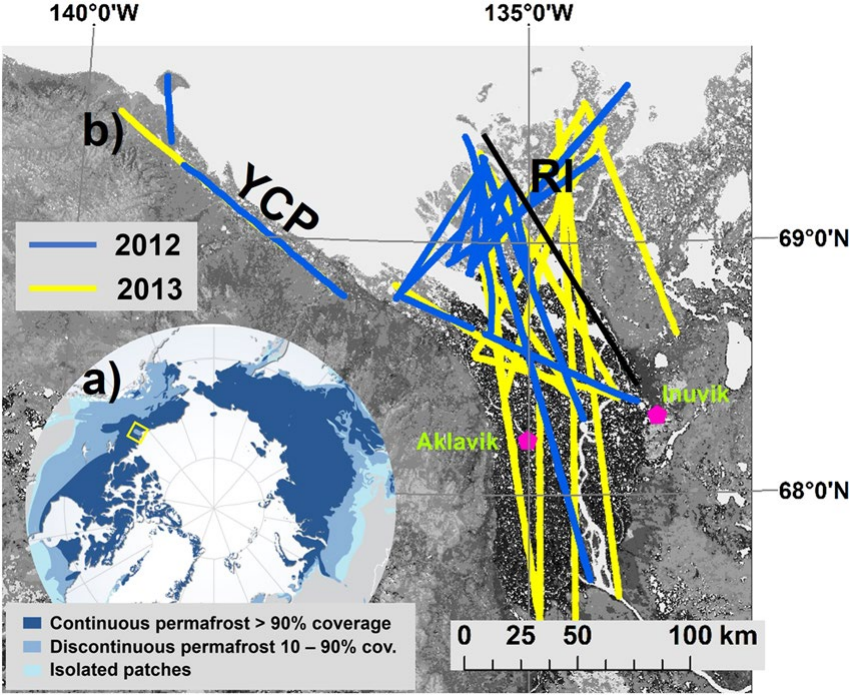
Study area and flight tracks. (**a**) Location of the study area and state of the permafrost (map modified after ref. 45), (**b**) flight tracks of two flight campaigns in 2012 and 2013 across the Mackenzie Delta, the adjacent Richards Island (RI) and the Yukon coastal plain (YCP). The black line marks the sharp transition between discontinuous permafrost in the delta to continuous permafrost on RI. The locations of the towns of Aklavik and Inuvik are shown for orientation. Data for background map from ref. 46. The map in Fig. 1b was created using ArcGIS software ArcMap 10.1 by Esri.

Among the largest amounts of oil and natural gas in the Arctic are likely stored in the Canadian Mackenzie Delta and its surroundings^10^. In this region, strong natural gas seeps have been described sporadically^17, 18^, some of which are fed by thermogenic CH_4_ (refs 17–19). The permafrost in the delta is of the non-Yedoma type, i.e. without the ice-rich, organic rich characteristics of Yedoma permafrost^20^. The permafrost state in our study area shows differences due to glaciation history and surface characteristics^21^. The delta itself is characterized by relatively thin (i.e. up to 100 m) and discontinuous permafrost as it was glaciated during the Pleistocene and waterbodies and channels are changing the surface and warming the ground^21^. The region east of the delta was unglaciated during the Pleistocene^22^ resulting in a sharp increase in permafrost thickness between the delta and Richards Island (marked with black line in Fig. 1b). On Richards Island the permafrost is up to more than 500 m thick and continuous^21^ (Fig. 1b). On the Yukon coastal plain the permafrost thickness reaches about 300 m. The soil organic carbon stocks in the study area are classified as medium to high soil carbon content with mainly 50–70 kg m^−2^ in the upper 1 m (ref. 7).

The overall aim of this study is to improve our understanding of geologic CH_4_ emissions on a regional scale in the Mackenzie Delta region, Canada (Fig. 1). Therefore, the purpose of this study is twofold: (i) to map the CH_4_ flux and its spatial variability at high spatial resolution and to derive the abundance of geologic CH_4_ emission hotspots and (ii) to assess the relative contribution of biogenic and geologic sources to the annual CH_4_ budget of the Mackenzie Delta region.

## Results and Discussion

We conducted airborne eddy-covariance (EC) measurements (cf. ref. 23) covering large areas of the Mackenzie Delta region during two extensive campaigns in July 2012 and 2013 (Fig. 1b).

**Figure 2:**
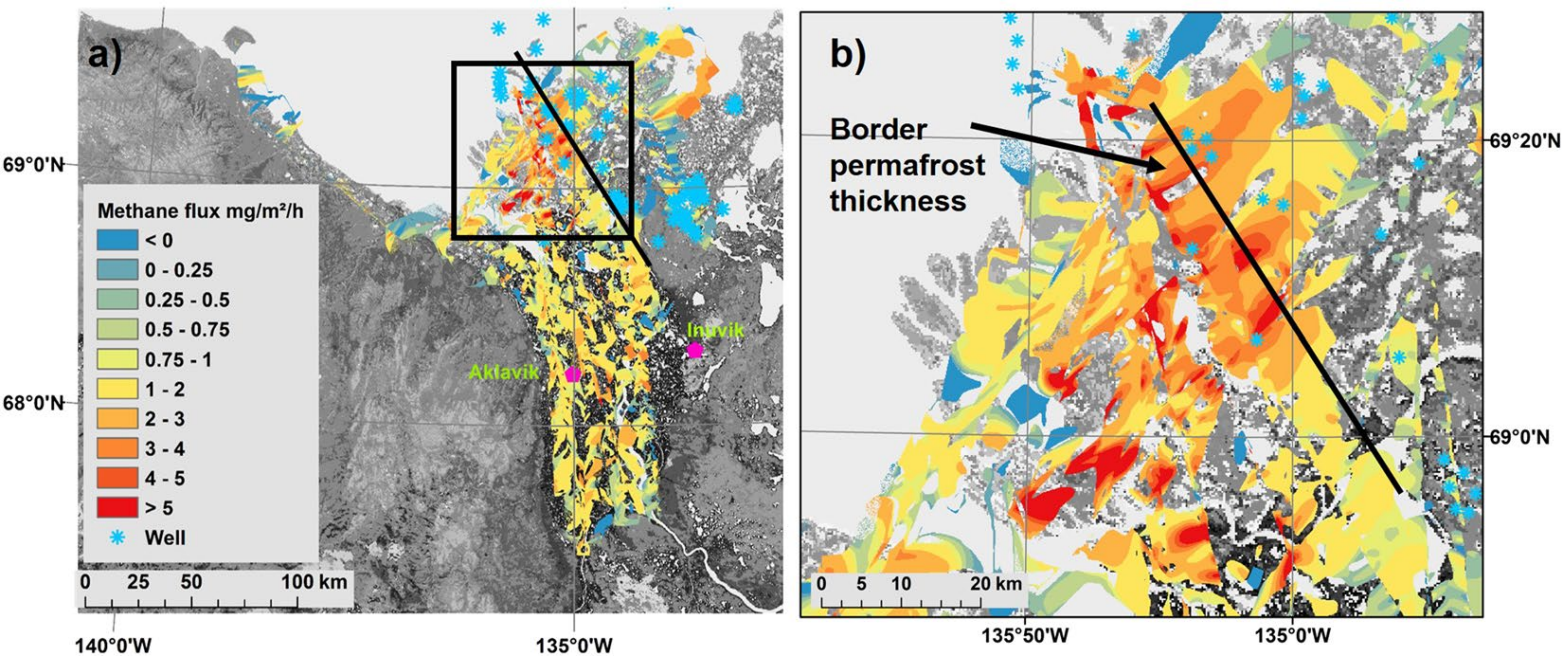
CH_4_ flux map with location of wells. (**a**) CH_4_ flux topography for both years containing data with a standard error <30%. Fluxes >5.0 mg m^−2^ h^−1^ are considered to be of geologic origin. Blue asterisks show the location of wells (oil or gas wells) derived from literature data. The locations of the towns of Inuvik and Aklavik are shown for orientation. (**b**) Magnification of the area with the highest CH_4_ fluxes marked with the black square in Fig. 2a. Legend as in Fig. 2a. West of the black line the permafrost is discontinuous and thin, east of it continuous and thick. Data for background map from ref. 46. The map in Fig. 2 was created using ArcGIS software ArcMap 10.1 by Esri. Methods for deriving the CH_4_ flux map are explained in chapters 4.2–4.5.

The CH_4_ flux map (cf. ref. 24) resulting from our measurements covers 10,000 km² at a spatial resolution of 100 m × 100 m (Fig. 2a). This CH_4_ flux map enables to detect spatial patterns in CH_4_ emission and to identify and localize CH_4_ emission hotspots in our entire study area. The median of all 2012 and 2013 CH_4_ flux data was 1.1 mg m^−2^ h^−1^, which corresponds to fluxes measured by the EC technique in similar ecosystems^25–29^. Most importantly, we found the largest emissions to be spatially stationary and temporally constant to <30% standard error, both within and between the two campaigns. A majority of high fluxes up to 14.7 mg CH_4_ m^−2^ h^−1^ (with a standard error of <30%) occurred as clustered peaks in the northern part of the study area and only inside the delta (Fig. 2) where the permafrost is thin and discontinuous. Biogenic CH_4_ emissions from arctic wetlands^25–29^ and lakes^30^ are typically lower and driven by changing meteorological and surface properties, making stationary, repeatedly observable emission peaks at this strength and spatial extent unlikely.

For lack of isotopic data, we used published biogenic CH_4_ flux data from EC flux tower measurements in the Arctic north of 61 °N for a conservative, approximate separation between biogenic emissions and strong geologic CH_4_ hotspots. The area of influence of tower based EC measurements, the so-called footprint, is of a comparable spatial scale as those of our airborne EC measurements, integrating over several hectares. Measurements with chambers and bubble traps, on the other hand, cover much smaller scales and do not readily compare with spatially integrated EC measurements. They were therefore not considered for the following threshold definition. The maximum daily biogenic CH_4_ flux from permafrost landscapes found in the literature is roughly 5.0 mg m^−2^ h^−1^ (e.g. refs 25–29; max. 4.58 mg m^−2^ h^−1^, ref. 28). At thermokarst margins of lakes with strong biogenic point sources, CH_4_ emission can reach 5.0 mg m^−2^ h^−1^ as well^13^. Individual hotspots of biogenic CH_4_ emission in lakes in carbon rich Yedoma type permafrost areas, can exceed that threshold^31^, but have an area of a few square metres, and thus are much smaller than the areas with CH_4_ emission peaks that we observed. These high emissions from lakes do occur on a seep scale and their signal would disappear among neighbouring areas with less CH_4_ emission, when we consider spatially integrated fluxes resulting from the EC method.

Therefore, we defined a flux of 5.0 mg m^−2^ h^−1^ as upper threshold for biogenic CH_4_ fluxes from arctic permafrost landscapes. We thus assumed that reoccurring emissions exceeding 5 mg m^−2^ h^−1^ independently of atmospheric or surface conditions, day, time or year of flight, as found in the northern Mackenzie Delta, were not of recent biogenic origin. Instead, we attributed them to deeper geologic sources that release CH_4_ through seeps, which can be related to taliks, faults or artificial pathways such as oil and gas exploration wells (Fig. 2).

Combined, the footprints of our 2012 and 2013 measurements comprise 9,754 km² excluding areas with a standard error >30%. We find that about 1% of the mapped area (116 km^2^), releases CH_4_ at rates exceeding 5 mg m^−2^ h^−1^. Single areas with these peak emissions are up to several square kilometres large. At this spatial extent, biogenic emissions from thermokarst lakes that exceeded this threshold have not been reported^30^. Single biogenic seeps in lakes would not result in an integrated signal that large.

We only found areas with high emissions within the northern part of the delta where the permafrost thickness is less than 100 m and the permafrost is discontinuous^21^ and therefore permeable for gas from the subsurface. In contrast, on the adjacent coastal plain and Richards Island, with their continuous and thick permafrost of up to 300 m and more than 500 m, respectively, such high emissions were not observed. We attribute that absence of emission peaks, despite established natural gas and oil deposits in the region (Fig. 2), to the thick impermeable permafrost in these areas. These CH_4_ reserves might eventually be emitted into the atmosphere if the permafrost cap becomes permeable due to thawing.

To estimate the contribution of geologic sources to the annual CH_4_ emissions of the study region, we divided the year roughly into a season with and without biogenic emission. For the following calculations, we did not account for potential biogenic emission from hotspots along thermokarst lake margins during winter time^6, 32, 33^, as they are weaker than geologic hotspots as described above. Biogenic emissions into the atmosphere from permafrost wetlands and lakes are largely limited to the growing or ice-free season, respectively, and the “zero curtain” period with soil temperatures near 0 °C (cf. ref. 9). Once soil temperatures drop permanently further during the cold season, the CH_4_ emissions decrease rapidly to close to 0 mg m^−2^ h^−1^ (ref. 9). We derived the period of potential biogenic CH_4_ emissions from soil temperatures in 10 cm depth from the North American Regional Reanalysis data^34^. In the particularly warm year of 2013, soil temperatures in the Mackenzie Delta exceeded −2 °C on about 165 days from end of May to beginning of November. We used this duration for biogenic emissions in the following back-of-the-envelope calculations. In contrast, we assumed that the emission of CH_4_ from strong geologic sources is constant throughout the year, as it is independent of recent microbial activity and causes sufficiently strong ebullition in water bodies to maintain pathways through the ice during winter^13^. Using the median fluxes of biogenic (0.84 mg m^−2^ h^−1^) and geologic (6.3 mg m^−2^ h^−1^) emissions, respectively, geologic CH_4_ contributes 3 Gg to the overall 35 Gg CH_4_ emitted within the covered area during the 165 days warm period. Annually, 38 Gg CH_4_ are emitted within this area, including 6.4 Gg CH_4_ from geologic sources. The geologic estimates are very conservative, first, because, fluxes <5 mg m^−2^ h^−1^ may also include a significant amount of CH_4_ from weaker geologic sources and, secondly, because extrapolating July biogenic fluxes, which are at or near a possible seasonal peak, into the shoulder seasons might overestimate the contribution of biogenic sources. Relative to their areal coverage, strong geologic sources that occur in 1% of the area thus contribute a disproportionally large fraction of at least 17% of the emissions to the estimated annual budget, making geologic sources 20 times stronger CH_4_ emitters than biogenic sources.

## Conclusions

Our study suggests, first, that thinning permafrost in a warmer climate may not only result in the frequently reported and discussed increased emission of biogenic CH_4_, but also in increased emissions of geologic CH_4_, that is currently still trapped under thick, continuous permafrost, as new emission pathways open due to thawing permafrost.

Secondly, in other arctic regions with natural gas and oil reservoirs that are currently capped under mainly continuous permafrost, e.g. the North Slope of Alaska or Siberia^10^, CH_4_ contributions from those geologic sources might need to be included when addressing future CH_4_ emissions under ongoing permafrost thaw.

Thirdly, our results indicate that geologic CH_4_ emissions may contribute strongly to the permafrost-carbon-climate feedback, especially in permafrost areas vulnerable to thawing and therefore warrant much more attention.

## Methods

### Experimental Setup

This study was performed in the Mackenzie Delta, which is the second largest arctic delta (13,000 km²), along the adjacent Yukon coastal plain towards Herschel Island, and on Richards Island to the north east of the delta (67°26′N–69°33′N, 133°22′W–140°5′W). The study area extended about 320 km from west to east and had a north-south extension of 240 km. The study period was from July 4^th^ to July 10^th^, 2012 (5 flight days, 44 flight tracks) and July 19^th^ to July 26^th^, 2013 (7 flight days, 40 flight tracks).

Measurements were conducted aboard the research aircraft Polar 5 of the Alfred Wegener Institute Helmholtz Centre for Polar and Marine Sciences (AWI). A 3 m nose boom including a 5-hole probe for measuring the 3D wind vector was mounted to the front of the airplane. Sample air was drawn from an inlet above the cabin at about 9.7 l s^−1^ and analysed in an RMT-200 (Los Gatos Research Inc., Mountain View, California, USA) in 2012 (CH_4_ concentration only) and a Fast Greenhouse Gas Analyser FGGA 24EP (Los Gatos Research Inc.) in 2013 (CH_4_, carbon dioxide and water vapour). Gas concentrations were recorded at 20 Hz, wind speed at 100 Hz. Additionally, the aircraft was equipped with an Inertial Navigation System (Type Laseref V, Honeywell International Inc., Morristown, New Jersey, USA), several Global Positioning Systems (NovAtel Inc., Calgary, Alberta, USA), a radar altimeter (KRA 405B/Honeywell International Inc., Morristown, New Jersey, USA) and a laser altimeter (LD90/RIEGL Laser Measurements Systems GmbH, Horn, Austria). Air temperature was measured with an open wire Pt100 in an unheated Rosemount housing, and air humidity with an HMT-330 (Vaisala, Helsinki, Finland) placed in a Rosemount housing. We derived the wind components u, v, and w with respect to the earth coordinate system using the method suggested by ref. 35. For the analysis only flux measurements derived after the warming up phase of the gas analysers (about 0.5 h) were used. Flight altitude was between 40–80 m above ground level, true airspeed was 60 ms^−1^. A height dependency of CH_4_ fluxes was not considered in our calculation. The atmospheric boundary layer (ABL) was determined with *in*-*situ* measurements of relative humidity and potential temperature during vertical profile flights at the beginning and end of each track. Flights above the ABL were excluded from the analysis.

### Flux calculation

The data were analysed in GNU R version 2.15.3 using an early version of the eddy4R software^36^. The analysis is based on 20 Hz dry mole fraction data. We calculated the dry mole fraction by using the humidity information from the Vaisala HMT-330 in 2012, and that from the FGGA itself for the 2013 data. Spectroscopic correction of the data was done following ref. 37. CH_4_ fluxes were calculated with a time-frequency-resolved version of the eddy covariance technique using vertical wind speed and CH_4_ concentration data^23^. The result was an *in*-*situ* observed space-series of the surface-atmosphere exchange of CH_4_ at 100 m spatial resolution. With a footprint model^38^ the CH_4_ emissions were related to the underlying surface. The data were quality controlled^39^ and only data up to flag 6 (steady state and integral turbulence characteristics test ≤100%), the upper threshold for data that are considered eligible for long-term measurements, were included in the analysis. This left 66957 flux observations (=6696 km of flight tracks), more than 90% of the original data, as input data for our study. In this study we did not apply spectral, vertical flux divergence, and storage term corrections.

### Flux topography calculation

To derive a flux map including CH_4_ fluxes from all flights, we used the flux values along the flight tracks and the corresponding footprints. The footprints provide (i) the information about the location, size and shape of the area influencing the flux measurement at the respective point, and (ii) the percentage contribution, i.e. footprint weight, from each grid cell to the combined flux signal observed by the aircraft. The weight of the contribution of each grid cell depends mainly on the distance from the measurement point, wind direction, and atmospheric stratification. That is, the footprint itself does not separate the strengths of individual sources. Instead, for each grid cell this is achieved by superimposing joint replicates of footprint weights and corresponding flux observations as the aircraft passes by. In result, the flux gradient around strong emitters appears continuous rather than representing the potentially discrete nature of geologic features. This last discretization step is achieved by applying the >5 mg CH_4_ m^−2^ h^−1^ thresholding.

We used flux topographies^24^ to derive the CH_4_ flux in each 100 m × 100 m grid cell using the sum of the products of CH_4_ fluxes and footprint weights divided by the sum of footprint weights covering that grid cell

$$\frac{{\sum }_{j}^{N}({\sum }_{i}^{M}{f}_{i,j}\ast \,{g}_{i,j})}{{\sum }_{j}^{N}({\sum }_{i}^{M}{g}_{i,j})}$$

with the number of flight tracks j … N, the number of footprint weights i … M that cover a grid cell during a given flight track, the CH_4_ flux f and the footprint weight g.

### Error calculation

In this study error sources include random measurement error, error in the wavelet calculation and in the footprint calculation. Standard error topographies were calculated following ref. 40. Grid cells with a standard error <30% (963843 grid cells; 57% of the data) were included in the analysis. The shape of the CH_4_ flux map presented in Fig. 2 is thus the result of the irregular shapes of the footprints caused by differences in wind speed and direction, areas not included within the footprints of our CH_4_ flux measurements, and the exclusion of these grid cells with a standard error >30%.

### Anthropogenic features

In the ArcGIS software ArcMap 10.1 by Esri we digitalized, georeferenced and compiled information from the geologic maps of refs 41–44 and marked locations of exploration gas and oil wells in Fig. 2.

### Estimating annual geologic and biogenic CH_4_ emissions

The upper threshold for biogenic fluxes was set to 5 mg CH_4_ m^−2^ h^−1^ based on maximum fluxes reported in the literature (e.g. refs 25–29). Emissions in grid cells exceeding this value were classified as geologic CH_4_. We derived the period of potential biogenic CH_4_ emissions from soil temperatures in 10 cm depth from the North American Regional Reanalysis (NARR) data^34^. Microbial activity and CH_4_ emission occurs in the warm period of the year and in the zero curtain period when soil temperatures fluctuate around 0 °C (ref. 9). CH_4_ emissions decrease close to zero, once the soil temperature drops well below 0 °C for the duration of the cold season^9^. Therefore, we defined the period between spring and autumn with soil temperatures exceeding −2 °C as period with biogenic emissions. In our study region this period lasted for about 165 days from end of May to beginning of November. In contrast, strong geologic sources are assumed to release CH_4_ to the surface continuously during 365 days a year if pathways exist. To calculate the emission from each of the two sources of CH_4_, we used the respective time periods, area size of occurrence and the median fluxes. We used the median flux of all measurements equal to or larger than 5 mg m^−2^ h^−1^ (6.3 mg m^−2^ h^−1^) for geologic emissions, and the median of those emissions smaller 5 mg m^−2^ h^−1^ (0.84 mg m^−2^ h^−1^) for biogenic emissions.

## References

[1] Schuur EAG (2015). Climate change and the permafrost carbon feedback. Nature.

[2] Chang RY-W (2014). Methane emissions from Alaska in 2012 from CARVE airborne observations. PNAS.

[3] Hodgkins SB (2014). Changes in peat chemistry associated with permafrost thaw increase greenhouse gas production. PNAS.

[4] Melton JR (2013). Present state of global wetland extent and wetland methane modelling: conclusions from a model inter-comparison project (WETCHIMP). Biogeosciences.

[5] Zona D (2009). Methane fluxes during the initiation of a large-scale water table manipulation experiment in Alaskan Arctic tundra. Glob. Biogeochem. Cycles.

[6] Walter KM, Zimov SA, Chanton JP, Verbyla D, Chapin FS (2006). Methane bubbling from Siberian thaw lakes as a positive feedback to climate warming. Nature.

[7] Hugelius G (2014). Estimated stocks of circumpolar permafrost carbon with quantified uncertainty ranges and identified data gaps. Biogeosciences.

[8] McGuire AD (2009). Sensitivity of the carbon cycle in the Arctic to climate change. Ecol. Monogr..

[9] Zona D (2016). Cold season emissions dominate the Arctic tundra methane budget. PNAS.

[10] Gautier DL (2009). Assessment of Undiscovered Oil and Gas in the Arctic. Science.

[11] Etiope G, Klusman RW (2010). Microseepage in drylands: Flux and implications in the global atmospheric source/sink budget of methane. Glob. Planet. Change.

[12] Romanovsky VE, Smith SL, Christiansen HH (2010). Permafrost Thermal State in the Polar Northern Hemisphere during the International Polar Year 2007–2009: a Synthesis. Permafrost Periglacial Process.

[13] Walter Anthony KM, Anthony P, Grosse G, Chanton J (2012). Geologic methane seeps along boundaries of Arctic permafrost thaw and melting glaciers. Nat. Geosci..

[14] Thornton BF, Geibel MC, Crill PM, Humborg C, Mörth C-M (2016). Methane fluxes from the sea to the atmosphere across the Siberian shelf seas. Geophys. Res. Lett..

[15] Shakhova N (2010). Extensive Methane Venting to the Atmosphere from Sediments of the east Siberian Arctic Shelf. Science.

[16] Etiope G, Klusman RW (2002). Geologic emissions of methane to the atmosphere. Chemosphere.

[17] Bowen, R. G., Dallimore, S. R., Côte, M. M., Wright, J. F. & Lorenson, T. D. Geomorphology and gas release from pockmark features in the Mackenzie Delta, Northwest Territories, Canada. In Proceedings of Ninth International Conference on Permafrost pp. 171–176, eds Kane, D. L. & Hinkel, K. M. Fairbanks, Alaska, Institute of Northern Engineering (2008).

[18] Osadetz KG, Chen Z (2010). A re-evaluation of Beaufort Sea-Mackenzie Delta basin gas hydrate resource potential: petroleum system approaches to non-conventional gas resource appraisal and geologically-sourced methane flux. B. Can. Petrol. Geol..

[19] Collett TS, Dallimore SR (1999). Hydrocarbon gases associated with permafrost in the Mackenzie Delta, Northwest Territories, Canada. Appl. Geochem..

[20] Strauss J (2013). The deep permafrost carbon pool of the Yedoma region in Siberia and Alaska. Geophys. Res. Lett..

[21] Burn CR, Kokelj SV (2009). The Environment and Permafrost of the Mackenzie Delta Area. Permafrost Periglacial Process.

[22] Taylor AE, Dallimore SR, Judge AS (1996). Late Quaternary history of the Mackenzie – Beaufort region, Arctic Canada, from modelling of permafrost temperatures. 2. The Mackenzie Delta – Tuktoyaktuk Coastlands. Can. J. Earth Sci..

[23] Metzger S (2013). Spatially explicit regionalization of airborne flux measurements using environmental response functions. Biogeosciences.

[24] Mauder M, Desjardins RL, MacPherson I (2008). Creating Surface Flux Maps From Airborne Measurements: Application to the Mackenzie Area GEWEX Study MAGS 1999. Boundary-Layer Meteorol.

[25] Sturtevant CS, Oechel WC, Zona D, Kim Y, Emerson CE (2012). Soil moisture control over autumn season methane flux, Arctic Coastal Plain of Alaska. Biogeosciences.

[26] Sachs T, Wille C, Boike J, Kutzbach L (2008). Environmental controls on ecosystem-scale CH_4_ emission from polygonal tundra in the Lena River Delta, Siberia. J. Geophys. Res..

[27] Wille C, Kutzbach L, Sachs T, Wagner D, Pfeiffer E-M (2008). Methane emission from Siberian arctic polygonal tundra: eddy covariance measurements and modeling. Glob. Change Biol..

[28] Friborg T, Christensen TR, Hansen BU, Nordstroem C, Soegaard H (2000). Trace gas exchange in a high-arctic valley 2. Landscape CH_4_ fluxes measured and modelled using eddy correlation data. Glob. Biogeochem. Cycles.

[29] Fan SM (1992). Micrometeorological Measurements of CH_4_ and CO_2_ Exchange Between the Atmosphere and Subarctic Tundra. J. Geophys. Res..

[30] Wik M, Varner RK, Walter Anthony K, MacIntyre S, Bastviken D (2016). Climate-sensitive northern lakes and ponds area critical components of methane release. Nat. Geosci..

[31] Walter Anthony (2016). Methane emissions proportional to permafrost carbon thawed in Arctic lakes since the 1950s. Nat. Geosci..

[32] Zimov SA (1997). North Siberian Lakes: A Methane Source Fueled by Pleistocene Carbon. Science.

[33] Sepulveda-Jauregui A, Walter Anthony KM, Martinez-Cruz K, Greene S, Thalasso F (2015). Methane and carbon dioxide emissions from 40 lakes along a north-south latitudinal transect in Alaska. Biogeosciences.

[34] Mesinger F (2006). North American Regional Reanalysis. Bull. Am. Meteorol. Soc.

[35] Lenschow, D. H. *Probing the Atmospheric Boundary Layer*. American Meteorological Society, Boston, Massachusetts, pp. 269 (1986).

[36] Metzger, S. *et al*. eddy4R: A community-extensible processing, analysis and modeling framework for eddy-covariance data based on R, Git, Docker and HDF5. *Geosci*. *Model Dev*. *Discuss*. (2017).

[37] Tuzson B (2010). Field intercomparison of two optical analyzers for CH4 eddy covariance flux measurements. Atmos. Meas. Tech..

[38] Kormann R, Meixner FX (2001). An analytical footprint model for non-neutral stratification. Boundary-Layer Meteorol..

[39] Foken, T. *Micrometeorology*. Springer-Verlag, Berlin, Heidelberg, pp. 308, ISBN: 978-3-540-74665-2 (2008).

[40] Gatz DF, Smith L (1995). The standard error of a weighted mean concentration – I. Bootstrapping vs other methods. Atmos. Environ..

[41] Dallimore, S. & Collett, T. S. Scientific results from the Mallik 2002 gas hydrate production research well program, Mackenzie Delta, Northwest Territories, Canada. Geological Survey of Canada, Natural Resources Canada, Bulletin 585, pp. 140 (4 sheets), 1 CD-ROM, doi:10.4095/220702 (2005).

[42] Dixon, J. (ed.) *Geological Atlas of the Beaufort*-*Mackenzie Area*. Natural Resources Canada, Ottawa, Ontario, pp. 178, doi:10.4095/207658 (1995).

[43] Harrison, J.C. *et al*. *Geological map of the Arctic/Carte géologique de l’Artique*; Map 2159A, Geological Survey of Canada, Natural Resources Canada, Ottawa, Ontario, doi: 10.4095/287868 (2011).

[44] Norris, D. K. *Geology*, *Aklavik*, *District of Mackenzie*: *A*-*Series 1517A*, Geological Survey of Canada, Natural Resources Canada, Ottawa, Ontario, doi:10.4095/109706 (1981).

[45] Brown, J., Ferrians, Jr. O. J., Heginbottom, J. A. & Melnikov, E. S. (eds) Circum-Arctic map of permafrost and ground-ice conditions. Washington, DC: U.S. Geological Survey in Cooperation with the Circum-Pacific Council for Energy and Mineral Resources. Circum-Pacific Map Series CP-45, scale 1:10,000,000, 1 sheet (1997).

[46] Latifovic, R., Olthof, I. & Pouliot, D. Land Cover Map of Canada 2005 from MODIS 250m. NRCan, ESS Program: Understanding Canada from Space. Ottawa, Ontario, Canada License: http://open.canada.ca/en/open-government-licence-canada (2008).

